# Noblesse Oblige

**Published:** 1923-05

**Authors:** 


### NOBLESSE OBLIGE

We are on the threshold of a new year which we believe will begin an era of great advancement in the dental instrument manufacturing business. Due to the merited success of "Udimcolite," we have every reason to believe that, in the near future, practically all dental instrument manufacturers will produce instruments that, they believe, have qualities similar to "Udimcolite."

We, as pioneers, having been the first to perfect a full line of non-corrosive operative instruments, naturally are flattered that our competitors feel it necessary to devote their energies towards bringing out an instrument to compete with "Udimcolite."

In developing "Udimcolite" we have had to overcome many obstacles. After continuous effort for several years which harbored many set-backs and disappointments, and involved the expenditure of much money for scientific research and experimentation, we are gratified to have perfected "Udimcolite" which combines all the qualities necessary to make an ideal instrument. It is reasonable to assume that other manufacturers developing a rustless and non-corrosive dental instrument will have to overcome similar obstacles and we feel that it will be some time before their product will have developed to a point anywhere near approaching ours in quality.

We, therefore, urge dealers to handle "Udimcolite" (the original Rustless and Non-Corrosive Instruments) which has gone through the stages of experimentation and is now a perfected product on the market to stay.

It is our intention to co-operate with our dealers in every way, especially in advertising. We hope to acquaint every dentist in the United States with the wonderful qualities and advantages of "Udimcolite" instruments.

We thank the dealers who have handled "Udimcolite" and appreciate the support they have given us, and hope that the handling of such a high grade instrument will, in 1923, bring them much satisfaction and profit.—Union Dental Instrument Mfg. Co., 831-35 Cherry St., Philadelphia.

